# Adverse effects of nanoparticles on humans

**DOI:** 10.1093/joccuh/uiaf021

**Published:** 2025-04-08

**Authors:** Slamet Wardoyo

**Affiliations:** Department of Environmental Health, Poltekkes Kemenkes Surabaya, Surabaya, Indonesia

**Keywords:** nanoparticles, occupational exposure, respiratory health, toxicity mechanisms

Dear Editor,

We appreciate the article entitled “Adverse effects of nanoparticles on humans” by Morimoto *et al* published in this journal. The article provides great insight into the adverse effects of nanoparticles on human health, especially for workers exposed in industrial settings. We would like to provide a response to this article with the aim of adding perspective as well as clarifying some relevant aspects in the context of nanoparticle toxicity.

This article effectively conveys that nanoparticles, including carbon nanotubes, welding fumes and organic substances, can have different health effects compared with micron-sized particles. Animal studies have shown that exposure to nanoparticles can cause lung inflammation, fibrosis and even tumors. This article also highlights the importance of considering the physicochemical properties of nanoparticles, such as size and shape, in determining their toxicity.^1^

The main strength of this article is its concise and comprehensive review of the existing literature on the health effects of nanoparticles. The article effectively highlights the gaps in our knowledge and the need for further research. In addition, the article discusses different types of nanoparticles and different exposures, such as inhalational exposure and intratracheal instillation. However, the article mainly focuses on animal studies, and human and epidemiological research is still limited. More in-depth studies of chronically exposed workers need to be conducted to determine the causal relationship between nanoparticles and various pulmonary diseases. Recent research by Schubauer-Berigan *et al*^2^ has shown an association between carbon nanotube exposure and increased respiratory symptoms in workers, but there is no strong evidence for the development of lung cancer or fibrosis in industrial worker populations.

In addition, it is important to consider how the size, shape, and chemical composition of nanoparticles affect their toxicity levels. A number of studies have shown that longer carbon nanotube fibers have a greater impact on lung inflammation compared to shorter fibers. In line with Morimoto *et al*’s findings, a study by Poland *et al*^3^ found that multi-walled carbon nanotubes (MWCNTs) longer than 15 μm have similar effects to asbestos in triggering lung inflammation and fibrosis.

We would also like to highlight that the cellular and molecular mechanisms underlying nanoparticle toxicity still require further exploration. One interesting hypothesis is that nanoparticles may trigger oxidative stress, chronic inflammation, and even genetic mutations that contribute to the pathogenesis of lung cancer. In addition, the interaction between nanoparticles and the immune system may lead to hypersensitivity reactions that could potentially exacerbate allergic respiratory diseases, as shown in several studies regarding increased allergic responses upon carbon nanotube exposure.

We would also like to highlight that regulatory aspects regarding occupational exposure to nanoparticles are still not fully established in many countries. Whereas Japan has set an occupational exposure limit for MWCNTs of 0.01 mg/m^3^, as mentioned in the article, other countries still do not have clear standards. Therefore, a global approach is needed in determining safe exposure limits for workers who are routinely exposed to nanoparticles.^4^

In conclusion, we greatly appreciate the contribution of Morimoto *et al* in presenting a broader understanding of the hazards of nanoparticles to human health. We hope that further research on the toxicological effects of nanoparticles, including more extensive epidemiological studies, can be conducted to clarify the health risks posed. In addition, we encourage the strengthening of regulations on nanoparticle exposure in the workplace to protect the health of workers.

Thank you to the authors for this valuable scientific contribution, and to the journal editors for providing space for constructive discussion. We believe that the study of nanoparticle toxicity will continue to grow and benefit global public health.
